# A CZT-based blood counter for quantitative molecular imaging

**DOI:** 10.1186/s40658-017-0184-5

**Published:** 2017-06-02

**Authors:** Romain Espagnet, Andrea Frezza, Jean-Pierre Martin, Louis-André Hamel, Laëtitia Lechippey, Jean-Mathieu Beauregard, Philippe Després

**Affiliations:** 1 Department of Physics, Engineering Physics and Optics and Cancer Research Center, Université Laval, Quebec City, G1V 0A6 QC Canada; 2 Department of Physics, Université de Montréal, C.P. 6128, Montréal, H3C 3J7 QC Canada; 3 Department of Medical Imaging and Research Center of CHU de Québec - Université Laval, Quebec City, G1R 2J6 QC Canada; 4 Department of Radiology and Nuclear medicine and Cancer Research Center, Université Laval, Quebec CityQC, G1V 0A6 Canada; 5 Department of Radiation Oncology and Research Center of CHU de Québec - Université Laval, Quebec City, G1R 2J6 QC Canada

**Keywords:** CZT, Gamma counter, Blood activity, Molecular imaging, PET scan, TAC

## Abstract

**Background:**

Robust quantitative analysis in positron emission tomography (PET) and in single-photon emission computed tomography (SPECT) typically requires the time-activity curve as an input function for the pharmacokinetic modeling of tracer uptake. For this purpose, a new automated tool for the determination of blood activity as a function of time is presented.

The device, compact enough to be used on the patient bed, relies on a peristaltic pump for continuous blood withdrawal at user-defined rates. Gamma detection is based on a 20 × 20 × 15 mm^3^ cadmium zinc telluride (CZT) detector, read by custom-made electronics and a field-programmable gate array-based signal processing unit. A graphical user interface (GUI) allows users to select parameters and easily perform acquisitions.

**Results:**

This paper presents the overall design of the device as well as the results related to the detector performance in terms of stability, sensitivity and energy resolution. Results from a patient study are also reported. The device achieved a sensitivity of 7.1 cps/(kBq/mL) and a minimum detectable activity of 2.5 kBq/ml for ^18^F. The gamma counter also demonstrated an excellent stability with a deviation in count rates inferior to 0.05% over 6 h. An energy resolution of 8% was achieved at 662 keV.

**Conclusions:**

The patient study was conclusive and demonstrated that the compact gamma blood counter developed has the sensitivity and the stability required to conduct quantitative molecular imaging studies in PET and SPECT.

## Background

Positron emission tomography (PET) and single-photon emission computed tomography (SPECT) are well-established molecular imaging modalities used in many fields of the biomedical sciences. They allow in vivo investigations of biological processes at the molecular level and provide valuable information on the onset and progression of diseases. The images obtained are based on a measurable number of nuclear disintegrations and, as such, are inherently quantitative. However, the quantitative nature of these modalities is usually dismissed, largely because tools and methods dedicated to quantitative imaging are lacking. Accurate quantification in PET and SPECT typically requires frequent assessments of blood activity, for example through manual sampling and subsequent measurements in a well counter. These methods however can be inaccurate and error-prone, and they expose the personnel to radiation and blood pathogen health hazards. The manual sampling methods might also be difficult to apply to tracers with a fast uptake.

Image-derived TACs are also possible and were investigated by several groups (see e.g. [1, 2] and references therein). These methods are less invasive, but they are not suitable for all anatomical sites, acquisition protocols and radiopharmaceuticals. They typically require the presence of a large blood vessel in the field-of-view (FOV), which is not always possible for dynamic acquisitions where the patient bed stays stationary over a particular FOV. Some image-based TAC also require corrections and sometimes suffer from methodological problems that hinder their widespread use [1]. Zanotti-Fregonara et al. for instance found that none out of eight image-based methods for TAC were reliable to estimate cerebral metabolic rate of glucose without resorting to blood sampling [2, 3]. Often, blood sampling is inevitable (to measure plasma vs whole blood TAC, for example) and remains the gold standard in all cases. Consequently, automated blood counters were developed for these reasons.

The majority of blood counters use a photomultiplier tube (PMT) coupled to a scintillation crystal such as BGO [4–8] or GSO [9, 10] to detect gammas. One device was build with an avalanche photodiode coupled to a LSO crystal [11]. This combination has the advantage of being compact compared to PMT-based solutions but suffers from a rather large temperature dependence. Other devices are based on PIN photodiodes and detect charged particles rather than gammas [12]. This design allows for compact devices but might suffer from low sensitivity as the tubing used can stop a significant fraction of electrons and positrons emitted from the blood. A microfluidic approach was recently used to overcome this problem [13].

In order to build a robust, sensitive and compact device, the cadmium zinc telluride (CZT) detector technology was used in this work [14, 15]. CZT detectors are known for their high energy resolution and stable operation (no temperature dependence). Although CZT will convert less gammas than BGO or GSO for a given volume, it has the potential advantage of requiring less shielding than PMT-based solutions and offers a better energy resolution. These advantages can result in more compact devices that can potentially improve quantification efforts in multi-isotope studies.

The objective of this work is to demonstrate the advantages of a CZT-based blood counter for PET and SPECT quantification. A compact prototype was designed and built to be used in a clinical setting. This paper will describe the main elements of the system and will present results regarding the detection sensitivity, the stability in time, the background noise and the minimum detectable activity (MDA). First results from a patient study are also reported.

## Methods

### General device features

**Fig. 1:**
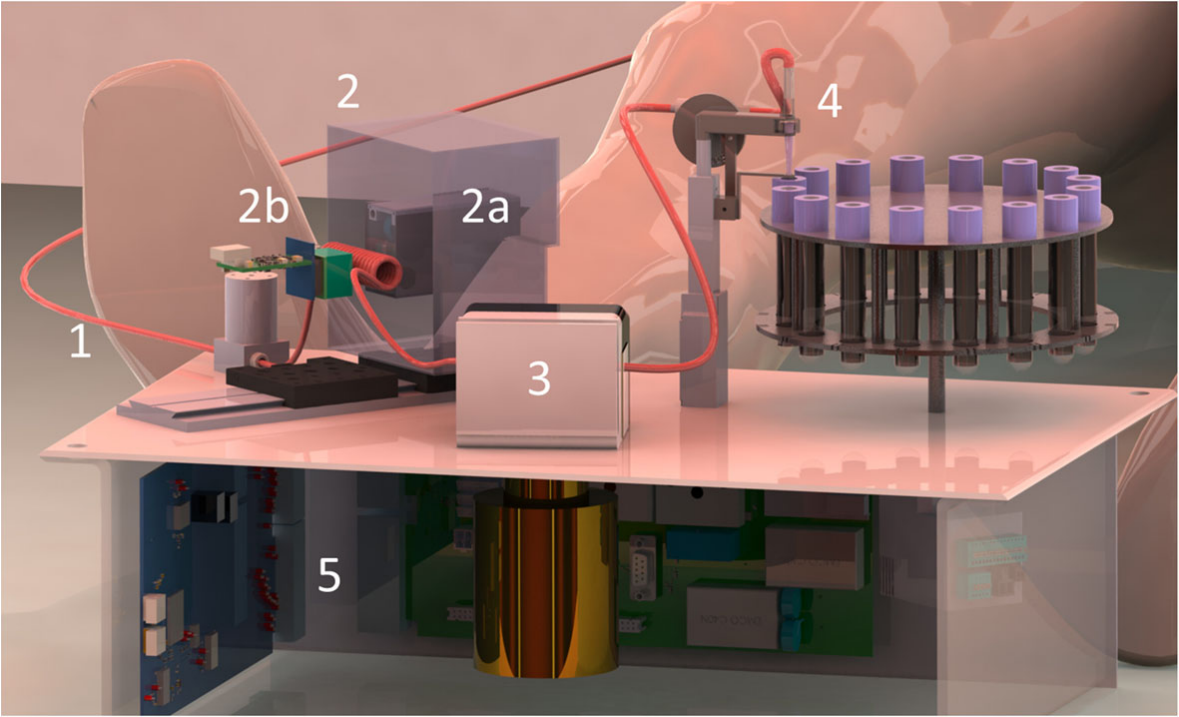
In this 3D rendering, the catheter *1* passes close to the gamma detection modules (*2a*, *2b* shown without shielding) then through the peristaltic pump *3*. A Y-connector allows to control the flow towards a waste container or a carousel holding evacuated tubes *4*. Acquisition boards are also shown *5*. For this work, a configuration with a single detector module *2a* was evaluated, shown here with its transparent-rendered shielding *2a*. The actual tungsten shielding is shown in Fig. 3

The device relies on gamma ray detection to determine the amount of activity in the blood. The system was designed to accommodate two detector modules, facing each other as shown in Fig. 1. These modules can be operated independently with the objective of increasing the sensitivity or in coincidence mode to reduce background counts. For the prototype evaluation, a single detector module was used, and therefore, coincidence counting was not performed. The prototype dimensions are 36 × 29 × 15 cm^3^ including all components: power supplies, electronic boards, motors and pump. The blood is withdrawn from the patient via a catheter (arterial or venous) connected to a peristaltic pump. A pump was dedicated to laboratory testing (model 313VDL, Watson Marlow, Falmouth, UK), while a second one, achieving lower withdrawal rates, was used in the patient study (model P625, Instech Laboratories, Plymouth Meeting PA, USA). The catheter delivers blood to the gamma counting system and then to a waste container. Figure 1 shows the overall organisation of components of the device. The blood withdrawal rate depends on the catheter size and can be set between 3 mL/min to 10 mL/min for the 313VDL pump and between 1 mL/min to 7 mL/min for the P625 pump.

The total amount of blood withdrawn from a patient should be kept as low as possible. McGuill et al. suggested to withdraw no more than 7.5% of the total blood volume [16]. It is therefore important to adjust the withdrawal parameters to the pharmacokinetic behaviour of a given radiopharmaceutical. The device built allows pre-programmed, variable pump rates in order to minimise the total amount of blood withdrawn for a given study while capturing the dynamics of uptake. Everett et al. have reported that in a 924 patients PET study, taking 117 to 137 mL of blood by arterial cannulation led to a single case of adverse effect (thrombotic occlusion) and concluded that the practise was safe, even if the catheter was in place for 5 h on average [17]. Zanotti-Fregonara et al. came to the same conclusions from their experience with more than 3000 patients [1].

The device must be located as close to the patient as possible to reduce activity dispersion along the catheter. Therefore, a design as compact as possible was sought for the device. The weight of the prototype was approximately 10 kg, including shielding. This allows the use of the device directly on the patient bed, thereby minimising the length of catheters required and reducing the effect of diffusion that affects the time resolution. The activity dispersion along the catheter can be estimated and corrected, typically by monoexponential deconvolution [18, 19] or step function calibration [20], but this was not implemented yet for the prototype device presented here. An access to radial blood with short catheters is more complicated in the case of brain studies where arms usually rest alongside the patient. In these cases, the system can be positioned at the feet of the patient with a longer catheter, at the expense of larger dispersion. For head studies with one or two bed positions, another possibility is to place the device on a cart beside the patient with a shorter access to the arm.

### Gamma detector

**Fig. 2:**
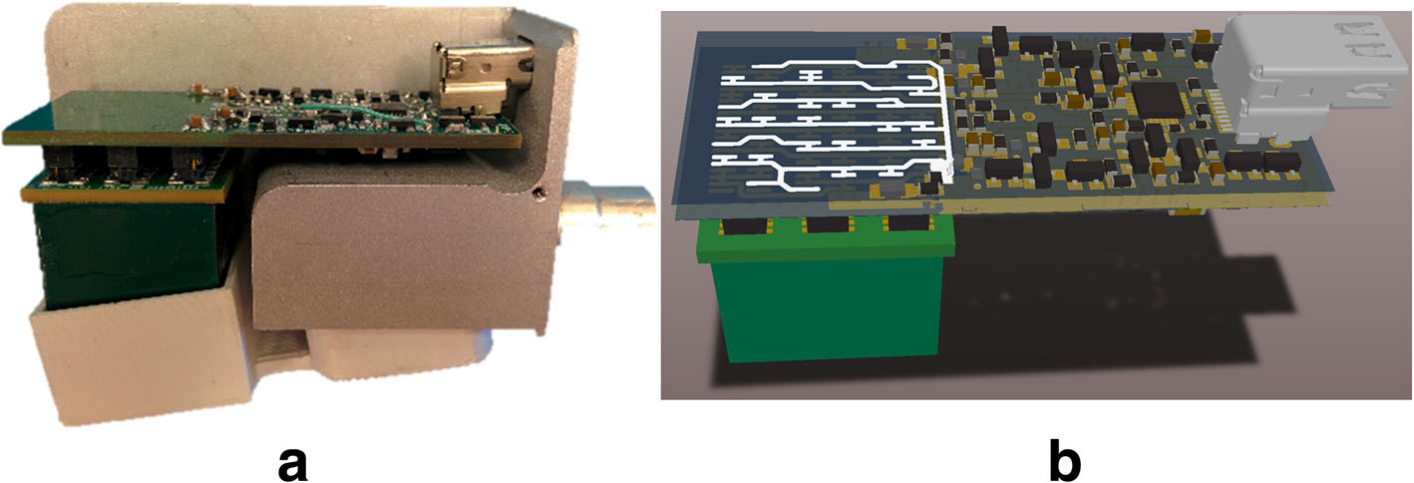
**a** An exploded view of the detector and preamplifier assembly. The CZT crystal is shown in *green* while the preamp board with the Mini DisplayPort connector is on *top*. The 3D-printed white plastic case is also shown and is used to isolate the high-voltage cathode and physically protect the CZT crystal. **b** A 3D rendering of the detection module showing one layer of the PCB and conductive tracks used to make a virtual coplanar detector from a pixelated detector

In order to fulfill compactness requirements, a CdZnTe (CZT) semi-conductor detector was selected. A 20 × 20× 15 mm ^3^ commercially available CZT crystal from Redlen Technologies (Saanichton BC, Canada) was chosen for the prototype, primarily for its large volume. Although this detector was designed primarily for imaging applications [21, 22] and has an 11 × 11 anode readout scheme, it provides the large detection volume required for the counting application developed here. Pixels on the detector are 1.22 mm in size deposited at a pitch of 1.72 mm. A grid of 0.1 mm is deposited 0.2 mm around the pixels, except at the edges of the pattern where it is 0.5 mm wide. For counting-only purposes, the readout pins of the 121 pixels were connected to obtain a pattern similar to a coplanar grid [23]. A custom-made front-end electronic on a six-layer PCB was used to create a virtual coplanar detector where pixels are connected column-wise, leading to a two-channel readout scheme where five columns are interleaved between six others and can be maintained at two different biases [15]. Figure 2 shows one layer of the PCB and conductive tracks used to make a virtual coplanar detector from a pixelated detector. This anode geometry with alterning columns maintained at different biases has the advantage of preserving energy resolution comparatively to a planar geometry and requires only two polarised anodes for readout [24, 25]. A custom-made charge sensitive preamplifier for each anode allowed the creation of a very compact board that also includes the appropriate routing of pixels to create a coplanar readout. The output of the preamplifier was fed to a dual-channel ultralow noise amplifier (AD8432, Analog Devices, MA, USA) which was used to obtain a differential signal that was then routed to the device’s main board by a Mini DisplayPort cable. The CZT crystal/preamplifier assembly was packaged in a compact custom-made 27 × 67 × 37 mm^3^ aluminium casing as shown in Fig. 2, along with 3D-printed pieces for accurate and reproducible positioning of all components. The weight of the detector module is 146 g.

**Fig. 3:**
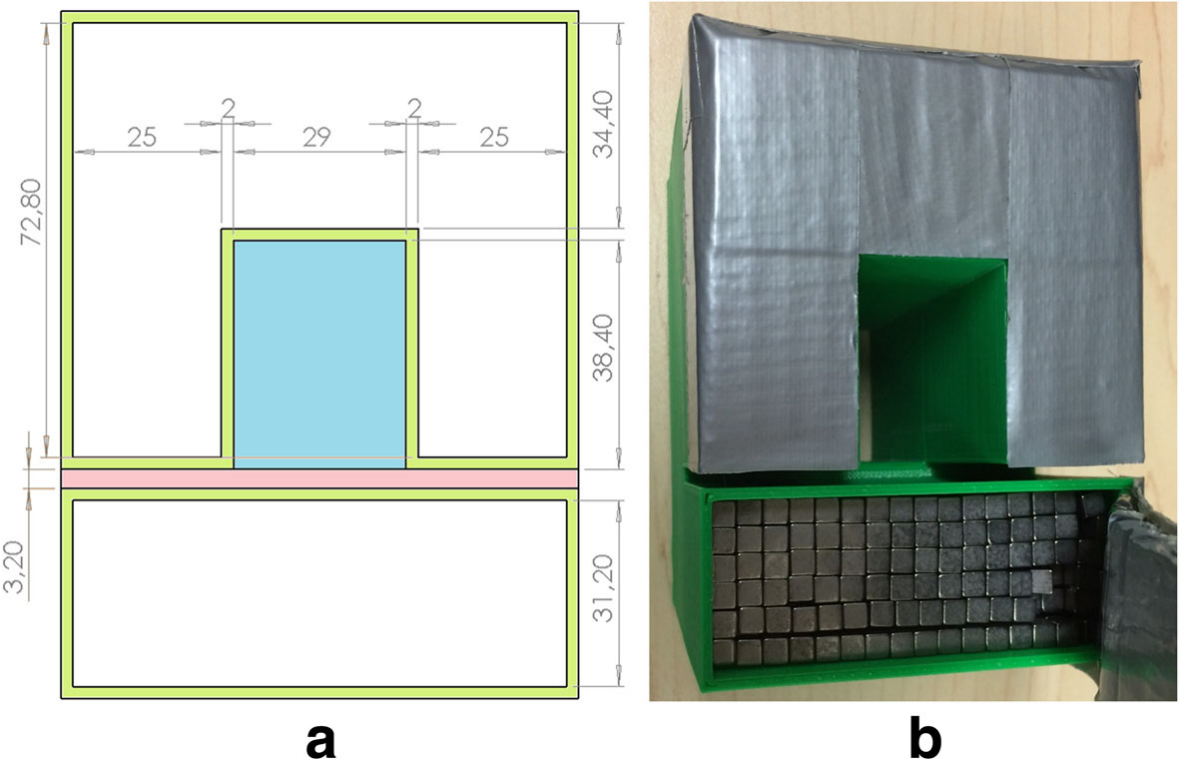
**a** Technical drawing in millimetre of the shielding container and **b** the actual shielding made of ABS plastic. The container was filled with 97% pure tungsten cubes. The detector assembly fits in the *blue zone* while the *red zone* shows the catheter space. The bottom of the *blue zone* (detector module enclosure) was shielded by 1 cm of tungsten (not shown) while the opening was not shielded to allow cables to exit (see Fig. 7)

**Fig. 4:**
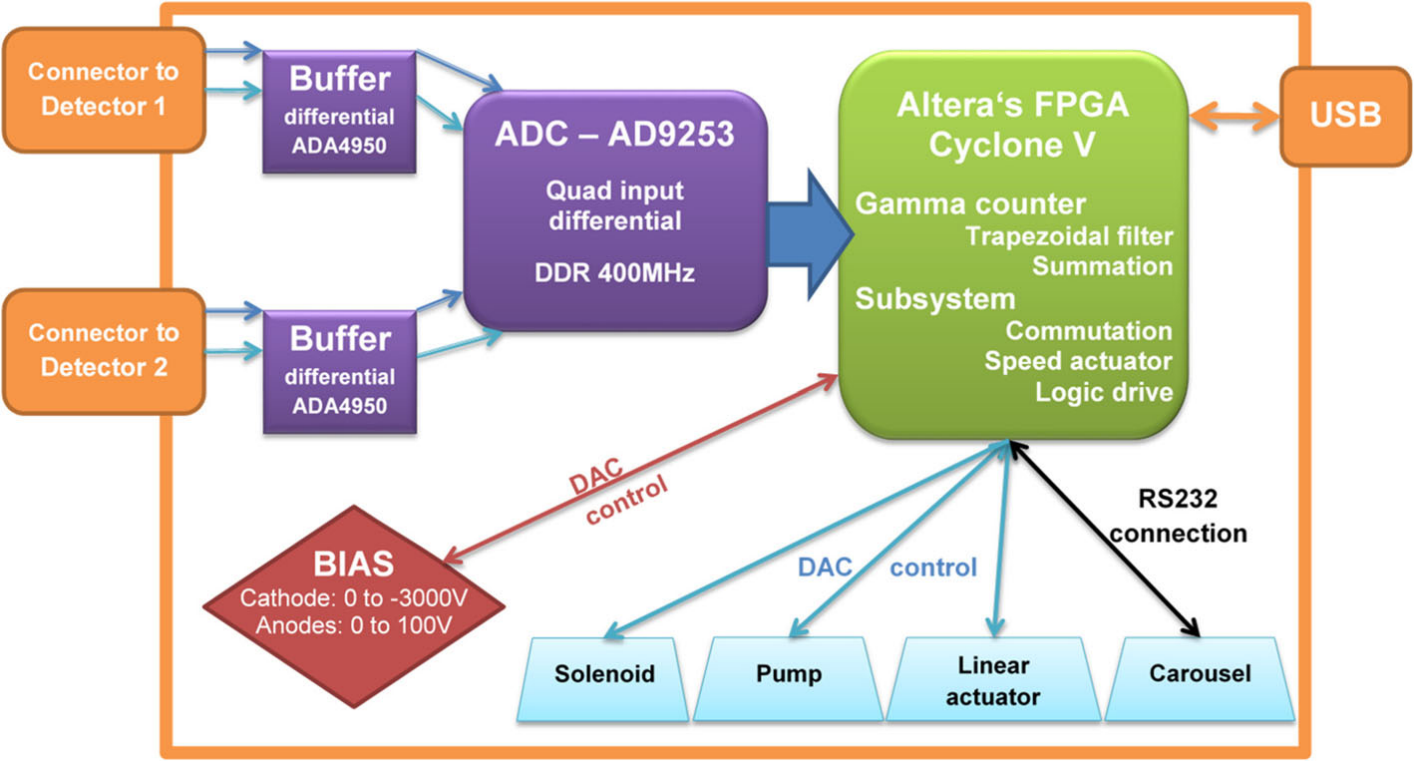
Signal workflow and connection diagram. The FPGA listens to the USB port and executes commands sent by the GUI

The detector assembly was shielded with 25 to 35 mm of 97% pure tungsten. A custom-made plastic container was 3D-printed and filled with tungsten cubes, as shown in Fig. 3. The container, which has a slit for the catheter, ensures reproducible positioning. A separate 3D-printed piece (not shown) slides in the slit and maintains the catheter in place. The length of the catheter exposed to the CZT detector is 29 mm. The detector was not shielded where the cables pass; this area was pointing towards the ceiling in the clinical experiments as shown in Fig. 8. In all cases, there was no direct unshielded line of sight between the CZT crystal and the main source of background radiation (the patient).

### Data acquisition

A field-programmable gate array (FPGA)-based (Cyclone V, Altera, CA, USA) circuit was designed to control the acquisition and the different subsystems of the blood counter. The FPGA chip allows a convenient handling of signals and components through the Nios II processor. The signal workflow is illustrated in Fig. 4. The analog signal from the detector-preamplifier assembly is digitised by a “free-running” quad, 14-bit analog-to-digital converter (ADC, AD9253, Analog Devices, MA, USA) and then routed to the FPGA where programmable filters and thresholds (shaping) are applied. More specifically, the two signals from the virtual coplanar detector are combined (weighted subtraction) on the FPGA to compensate for charge trapping, as described by Luke et al. [23, 26]. A thorough characterisation of the detector, not reported here, allowed to set optimal operation parameters of this anode configuration. A trapezoidal filter was applied on the resulting signal, and the maximum was extracted. Counts exceeding 70 keV were stored in memory while the FPGA is waiting for calls by the host program via a USB connection.

### Graphical user interface

**Fig. 5:**
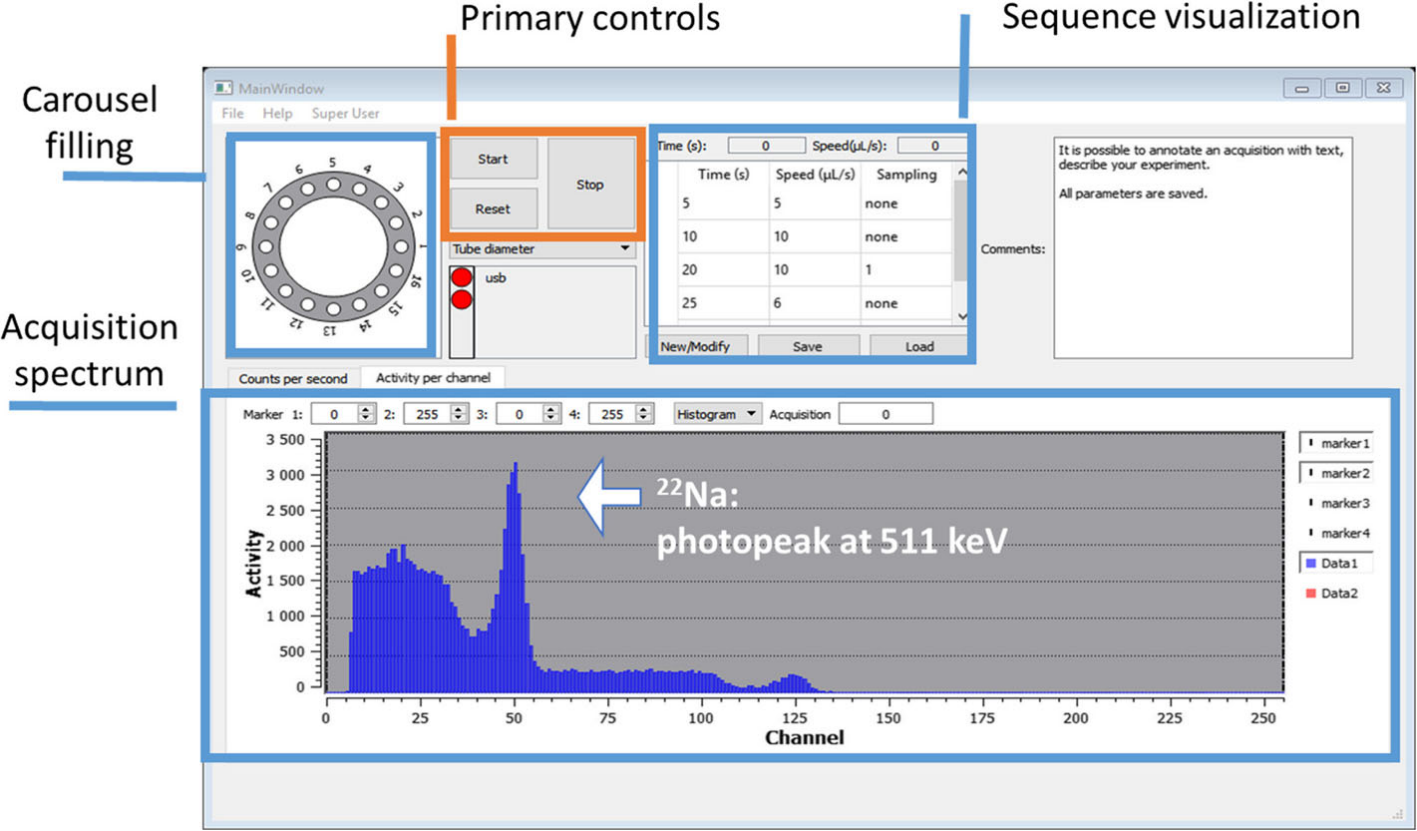
Screenshot of the GUI, showing the various controls and visualisation elements and a typical ^22^Na acquisition

The host program runs on a PC and has a graphical user interface (GUI) implemented with Qt (version 5.3, Helsinki, Finland). The GUI, shown in Fig. 5, can display the detected counts per second for two energy windows and two detector modules. The centre panel gathers the primary controls that allow an acquisition to start, stop and reinitialise. The information on the acquisition sequence is also shown in the central panel. It defines the pump rates and acquisition duration. Sequences can be programmed, saved and reused. The GUI also shows a visual representation of a carousel where discrete blood samples can be packaged in evacuated tubes for further analysis. For this work, the packaging feature of the device was not used.

The bottom part of the GUI shows either a graph of the activity as a function of time or an energy histogram of detected events, depending on which tab is selected. Four markers can be positioned to define two energy windows, allowing for example dual-isotope studies. The energy windows selected are applied to the activity displayed in the associated tab.

The GUI has a *user* mode for regular use and a *superuser* mode for development. The *superuser* mode allows a manual control of the device and real-time programming of pump rates, motors and data processing parameters. The *user* mode is meant to be used in a clinical setting and therefore allows the definition and use of pre-programmed acquisition sequences. For safety reasons, it is possible to bypass a sequence with manual control of the peristaltic pump or the detector.

### Device characterisation

For all acquisitions, pulse height histograms with 256 bins were obtained. The histograms were energy-calibrated with a ^137^Cs source of 32.7 kBq, and the energy threshold was set to 110 keV for all acquisitions. An estimation of energy resolution was obtained by fitting a Gaussian on the photopeak (662 keV) of the energy spectrum.

#### Stability over time and catheter positioning reproducibility

**Fig. 6:**
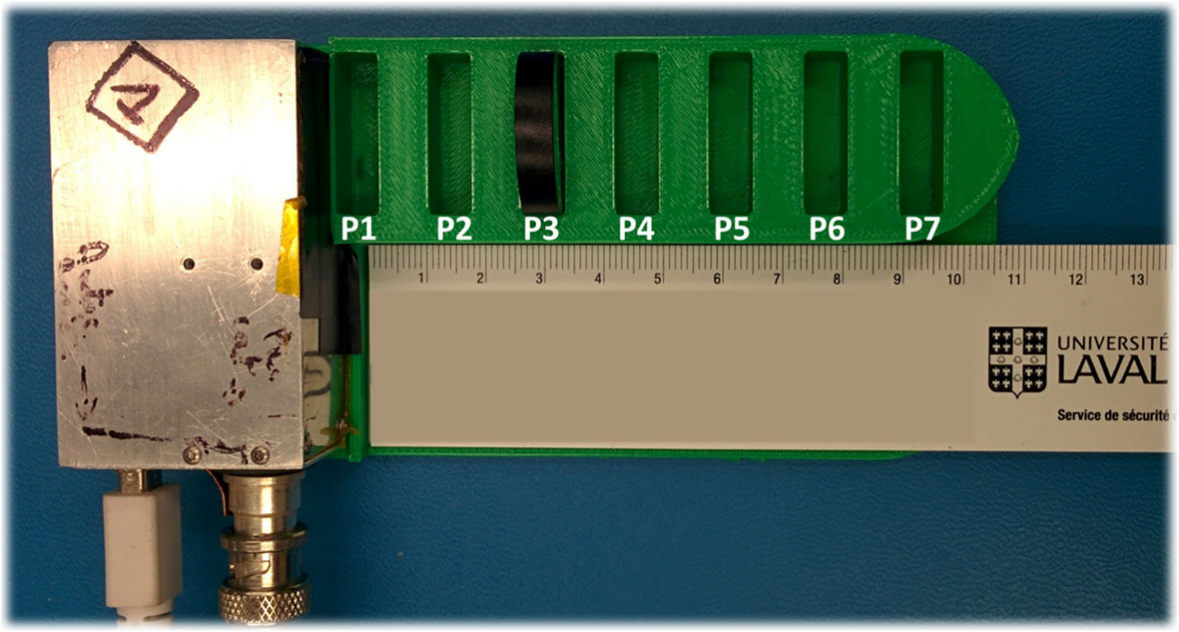
Positioning template for the ^137^Cs source

Two series of tests were performed to evaluate the stability of the detector over time, both conducted with a ^137^Cs source (half-life of 30.17 years). The first one was used to verify that there is no drift in count rates over a period of 6 h with an integration time per sample of 5 s. The second test consisted in performing 19 acquisitions of 3 min at random times over a period of 3 weeks with a counting time per sample of 1 s. A 3D-printed template, shown in Fig. 6, was used to ensure reproducible positioning of the source relative to the detector. The template allows positioning of the source every 15 mm from the detector, with the closest position at 3 mm.

**Fig. 7:**
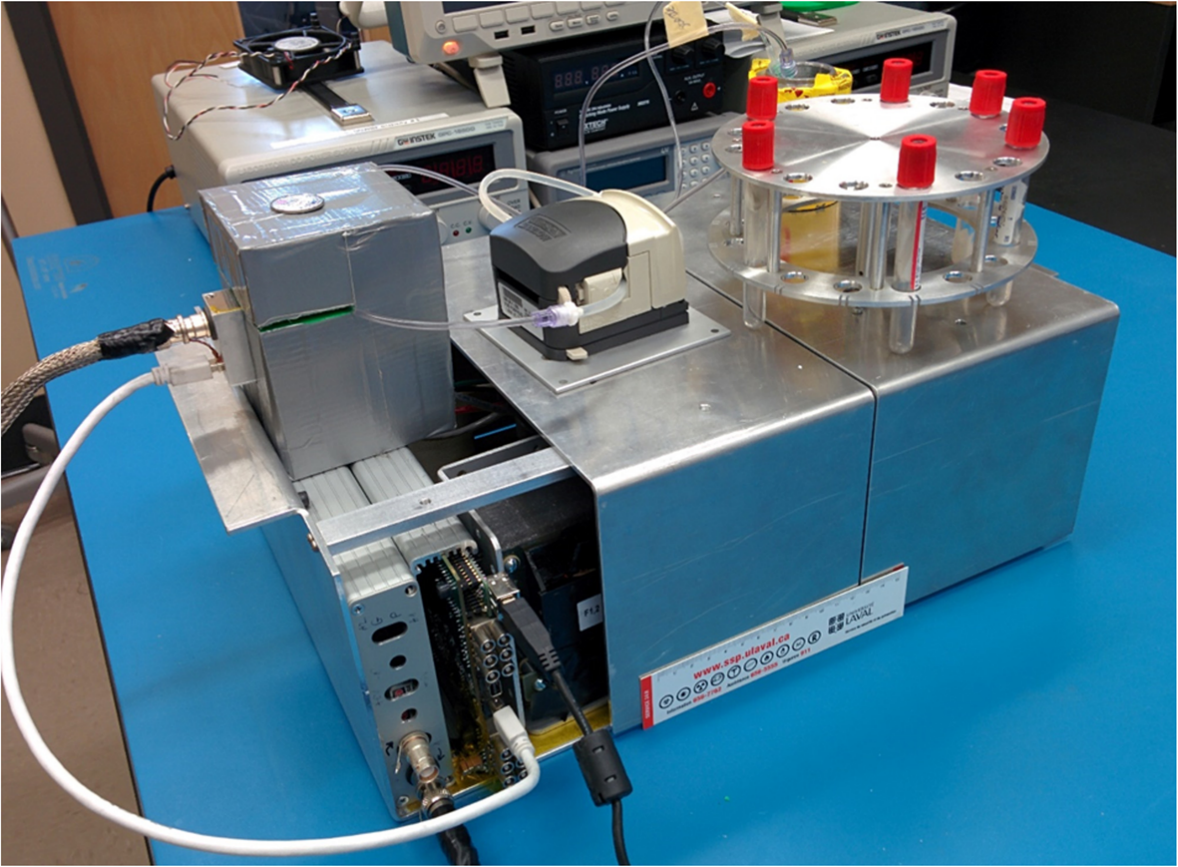
Setup used for calibration with circulating FDG

**Fig. 8:**
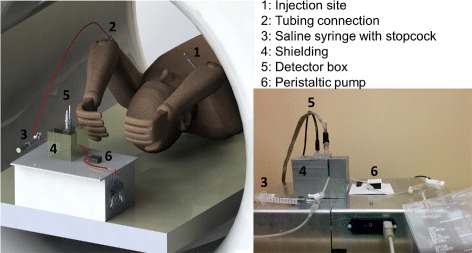
Setup used to extract time-activity curves during clinical PET studies. (*Left*) 3D rendering and (*right*) photograph

The first test over the 6 h acquisition was analysed by fitting the data to a linear function. A *χ* ^2^ test was used to verify that the data was Poisson-distributed, as expected.

In the second experiment, an analysis of variance (ANOVA) was performed to determine if the 19 acquisitions over 3 weeks belonged to a distribution with identical parameters (mean and variance).

Another experiment was conducted to verify that the catheter can be positioned in a reproducible manner each time. For a catheter filled with FDG, the catheter was removed and repositioned 20 times and counting was performed for 30 s with a counting time per sample of 1 s. Counting rates were decay-corrected, and an ANOVA was performed to assess reproducibility of catheter positioning. All statistical tests were performed with *R* (version 3.2.1).

#### Minimum detectable activity

The minimum detectable activity (MDA) is a crucial characteristic of the device; the counter must detect a small number of counts per second in the relatively high background of a PET scan room. This can be achieved through adequate shielding, coincidence counting or a combination of both. In this work, shielding only was used but the acquisition system of the device was designed with coincidence counting as an optional feature. Typically, the blood activity is lower than 500 kBq/ml at the maximum of the input function for fluorodeoxyglucose (FDG) [7].

The MDA, in units of kilobecquerel per millilitre, is defined at a 95% confidence interval by [27]:

1 $$ \text{MDA}= \frac{4.65\sqrt{N_{B}}+2.71}{fTs}  $$

where *T* is the counting time per sample, *N*_*B*_ is the number of background counts recorded during *T*, *f* is a factor of radiation yield per disintegration (*f* = 0.967 here) and *s* is the sensitivity of the detector as obtained by calibration. The sensitivity—the ratio of recorded count rate and activity concentration—was obtained with a 1.58-mm-inner-diameter catheter filled with 52.5 kBq/ml of FDG. The counting time per sample *T* used for MDA determination was 3 s, but it can be adjusted between 1 and 30 s to optimise the MDA as a function of background and activity level in the catheter. Figure 7 shows the prototype with FDG circulating in a catheter.

Background counts were measured in a realistic environment, i.e. in a PET scanner room at 1 m from a patient injected with 275 MBq of FDG 40 min prior to the experiment. The background count rate was averaged over a 3 min acquisition. The experiment was repeated with and without the tungsten shielding to estimate its efficacy.

#### Dispersion

To evaluate the activity dispersion in the tubing used, a step function study was conducted. A three-way valve was added at the end of the tubing (inner diameter of 1.58 mm) in a setup similar to the one used by Munk et al. [20]. Two vials were connected to the valve, one filled with water and the other with a mixture of water and FDG. Measurements were performed at two pump rates (2 and 4.5 ml min ^−1^) and two tubing lengths (80 and 45 cm). The rising part of the step was modelled by an exponential function, *f*(*t*) = *A*(1− exp−*t*/*τ*) for each case [19].

### PET study

The use of the device in real conditions is essential to verify that it meets clinical requirements and workflows. For this purpose, the device was tested in a clinical setting with prostate cancer patients undergoing dynamic ^18^F-fluoromethylcholine (FCH) PET studies. As shown in Fig. 8, the patient was in supine position with his arms positioned above his head. Two venous accesses were installed, one in each arm. FCH was injected in the left arm while blood was withdrawn from the right arm (18 gauge needle). Tubing of 76 cm with an inner diameter (ID) of 2.54 mm was connected between the patient and a stopcock. The stopcock interfaced a saline syringe and the tubing (ID of 1.58 mm) going to the P625 pump, the detector module and then the waste container. The blood withdrawal rate was set to 2 ml/min for the acquisition. The patient was injected with a standard activity of 4 MBq/kg for a total of 355 MBq. The injection was performed within 3 s. The pump was started approximately 1 min before the beginning of the acquisition, defined as the time where the FCH is injected while the PET acquisition and the gamma counting are started simultaneously. The dynamic PET scan with a field-of-view centred on the prostate lasted 600 s. To compensate for the transport delay of the blood in the tubing and to extend gamma counting time, 125 s were added to the gamma counting acquisition.

## Results

### Stability over time and catheter positioning reproducibility

**Fig. 9:**
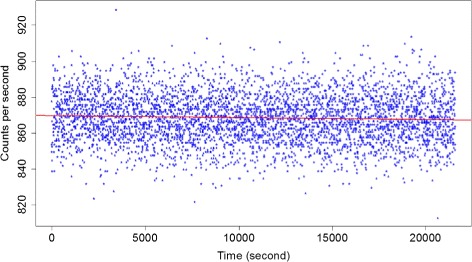
Acquisition with a ^137^Cs source over 6 h and linear fit through the data

Figure 9 shows the count rate as a function of time over a 6-h acquisition. A linear fit through the data yields a slope of 0.01%. This observed slope is not statistically significant, and the error on the slope of ± 0.05% was retained.

The data is Poisson-distributed as expected, with a reduced *χ* ^2^ value of 1.02.

**Fig. 10:**
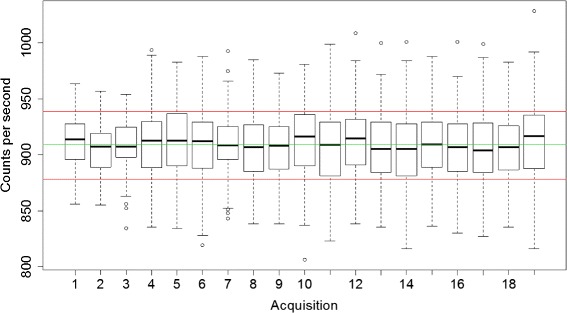
Box-and-whisker plot representation of nineteen 3-min measurements over 3 weeks, showing median values, upper and lower quartile values (*boxes*), minimum and maximum values excluding outliers (*whiskers*) and outliers (1.5 times the upper/lower quartiles shown as points). The *green line* represents the mean value of all data, while the *red line* represents one standard deviation from the mean

Figure 10 shows a boxplot representation of the 19 acquisitions of 3 min taken over a period of 3 weeks. The ANOVA analysis yielded a *p* value of 0.195, suggesting that the count rates observed over 3 weeks have the same mean as expected.

**Fig. 11:**
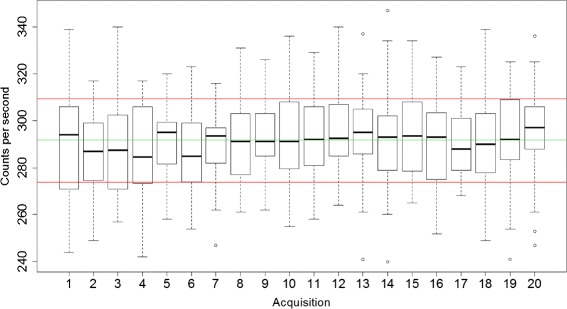
Box-and-whisker plot representation of twenty successive repositioning of the catheter, which is kept in place by a 3D-printed piece. The *green line* represents the mean value of all data and the *red line* represents one standard deviation from the mean

Figure 11 shows the results of the catheter repositioning experiment. A *p*=0.16 value was obtained for the ANOVA, suggesting that the positioning is reproducible.

### Minimum detectable activity and energy resolution

The sensitivity *s* obtained through calibration was 7.1 cps/(kBq/mL) for ^18^F. In the PET scanner room at 1 m from an FDG-injected patient, the measured background counts were 40 cps and 1500 cps with and without tungsten shielding respectively. For a 3 s sampling time, this yields a MDA of 2.5 kBq/ml with the tungsten shielding, which provides approximately a 30-fold reduction in background counts. The measured energy resolution of the detector was 8% at 662 keV.

### Dispersion

**Fig. 12:**
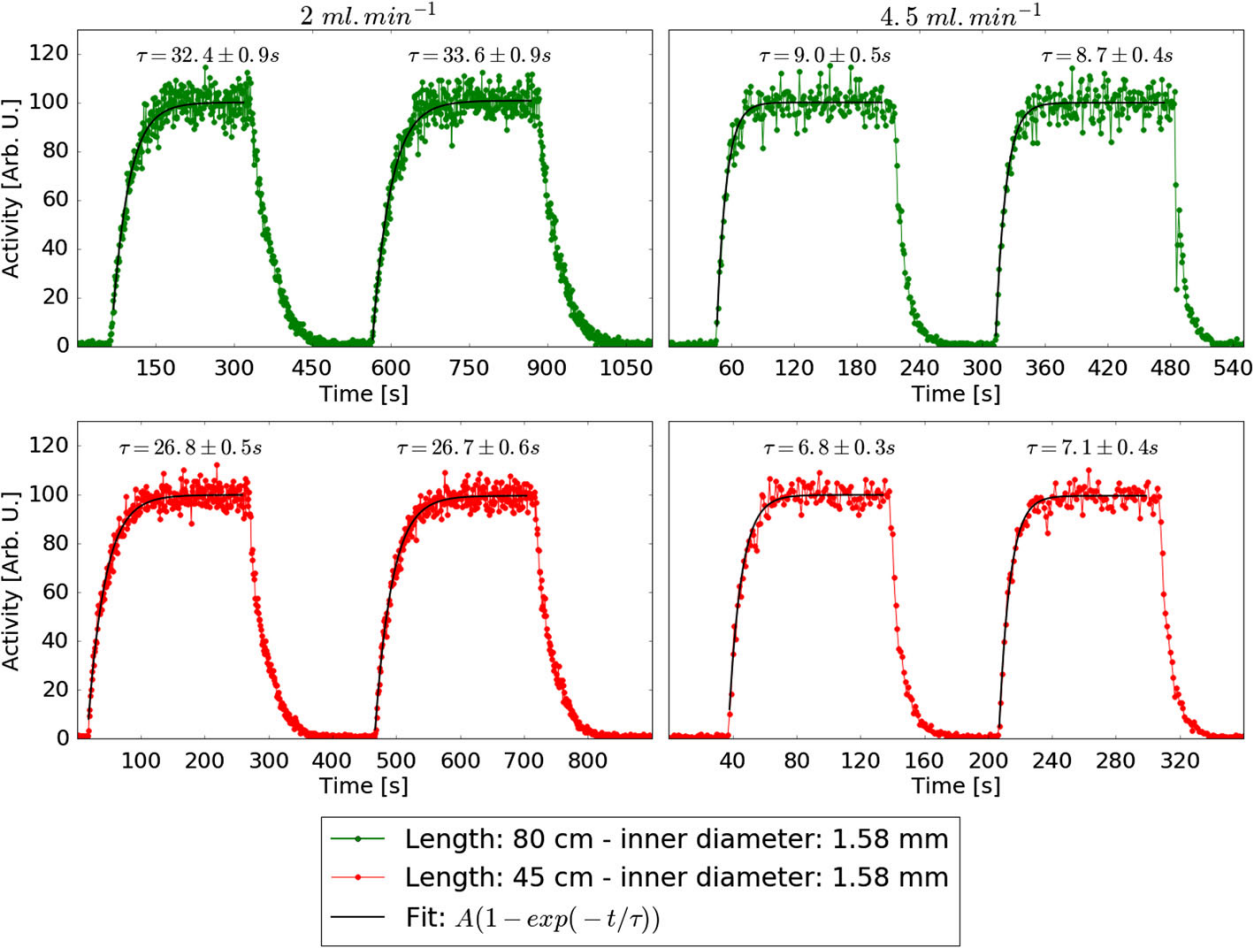
Step functions for different flow rates and tubing lengths

Figure 12 shows the step functions and related fits for the flow rates and tubing lengths used. The time constant *τ* extracted from the fits can be used to correct the TAC curves obtained, provided the experiment is conducted in the same conditions (liquid and radiopharmaceutical involved, tubing and pump rate used) [20].

### PET study

**Fig. 13:**
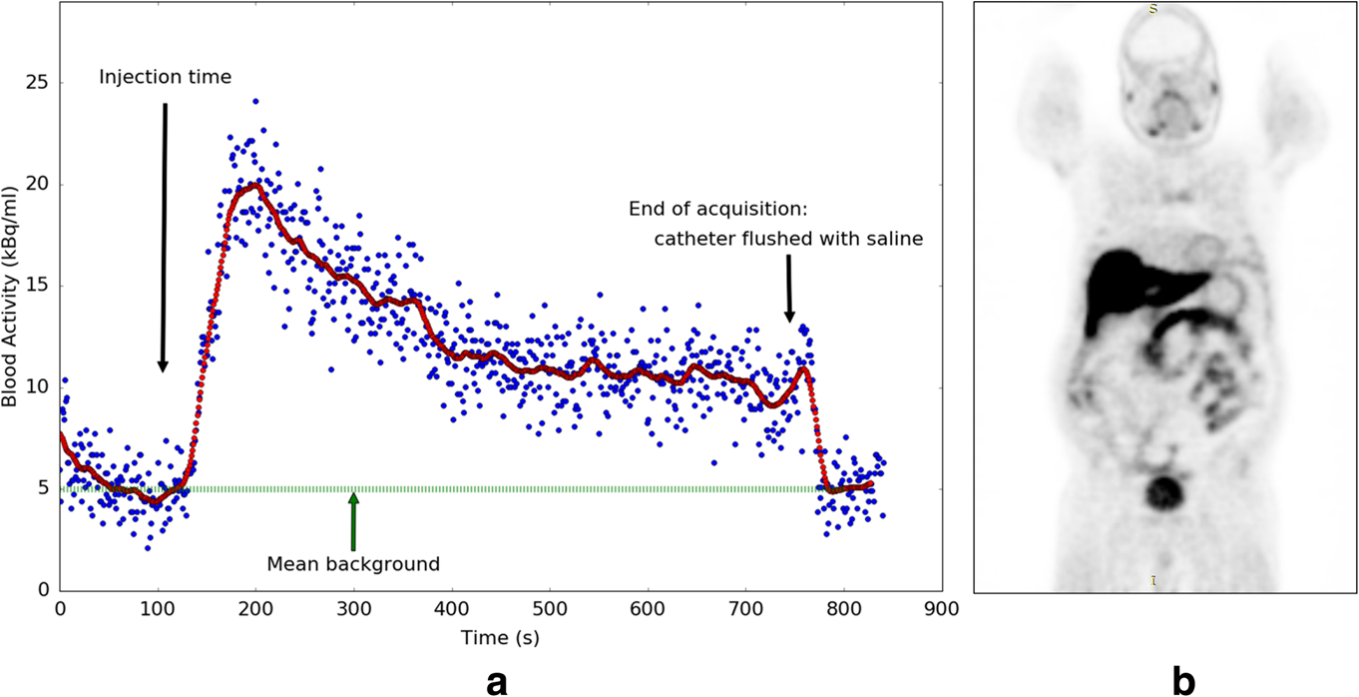
**a** Time-activity curve not corrected for dispersion, where the injection time corresponds to the beginning of the acquisition time corrected by 125 s. The dispersion is relatively large due to the long catheter used in this proof of concept study. **b** Dynamic PET image of the patient with attenuation correction

Figure 13 shows the PET image of the patient as well as the TAC in kilobecquerel per millilitre, as corrected by the calibrated system sensitivity but not for activity dispersion in the tubing. The measured blood activity was well above the background generated by the patient body, measured at 5 kBq/ml with the catheter filled with saline before and after the passage of blood.

## Discussion

The results show that the device fulfills compactness, stability and sensitivity requirements for a typical usage in molecular imaging. The measured sensitivity of 7.1 cps/(kBq/mL) translates into a MDA of 2.5 kBq/ml in a PET scanner room, an environment with a high background. This is well below the 500 kBq ml ^−1^ concentration typically encountered in blood for FDG studies [7]. The detector system count rate behaviour was shown to be linear below 500 kBq ml ^−1^, while obeying a non-paralysable model up to 2.5 MBq ml ^−1^ where dead time accounts for a 10% count loss [15].

This MDA value corresponds to a sampling time of 3 s, and it can be lowered by a factor 3.2 by increasing the sampling time to 30 s as the activity level decreases in the blood. The prototype allows such sequence programming through its GUI.

The device demonstrated high stability with a drift in count rates lower than 0.05% over 6 h. Random checks over 3 weeks also suggested high reliability. This robustness is a typical advantage of solid-state detectors over scintillators/PMT assemblies that might require periodic recalibration.

Table 1 reports a non-exhaustive list of characteristics of blood counters reported in the literature. Direct comparisons are not always straightforward as some devices were built for animal use only and some are based on the detection of positrons. However, the detector presented here exhibits performances similar to other detectors, while the overall prototype being relatively more compact than other designs.

**Table 1:** Comparison of real-time blood counters

	Detector				Technical characteristics						
	Type	Size^a^ (mm)	Number	Shielding (mm), material	*β* ^+^/*γ*	Sensitivity (^18^F) (cps/(kBq/ml))	MDA (^18^F) (kBq/ml)	Absolute efficiency (%)	Energy window	Time drift	Energy resolution
This work	CZT	20 × 20 × 15	1	30, W	*γ*	7.1	2.5	10.5% at 511 keV	110–1050 keV	<0.05% /h	8% at 662 keV
Eriksson et al. [5]	BGO/PMT	45 × 45 × 45	1	60, Pb	*γ*	25.5	0.37	–	350–650 keV	<1%	20%
Votaw and Shulman [6]	BGO/PMT	50 × 25 × 25	2	16, Pb	*γ*	7.3	–	6.9% at 511 keV	>350 keV	–	–
Boellaard et al. [7]	BGO/PMT	*D* = 60 *L* = 60	1	60, Pb	*γ*	23	–	–	30% of 511 keV	<3% /h	–
Kudomi et al. [9]	GSO/PMT	20 × 20 × 12.9	4	20, Pb	*γ*	>2.6	–	6.2% at 511 keV	>450 keV	–	11% at 511 keV
Twilite II (swisstrace)[28]	LYSO	–	2	–	*γ*	2.4	–	–	–	–	–
Laymon et al. [8]	BGO/PMT	*D* = 25 *L* = 25	2	–	*γ*	11 (^15^O)	–	19% at 511 keV	400–1360 keV	<0.6%	–
Breuer et al. [11]	LSO/APD	50 × 40 × 30	2	40, W	*γ*	–	–	18.5% at 511 keV	350–1000 keV	<1%	22% at 511 keV
Yamamoto et al. [10]	GSO/PMT	10 × 20 × 0.5	4	4, W	*β* ^+^/*γ*	–	–	8.6% (^18^F)	>80 keV	–	18.5% at 511 keV
Convert et al. [12]	Si photodiode	30 × 3	1	<13, W	*β* ^+^	–	23	7.1% (^18^F)	–	–	–
Convert et al. [13]	Si photodiode	16 × 2	1	–	*β* ^+^	–	23–51	39% (^18^F)	–	–	–
Roehrbacher et al. [29]	LYSO/PMT	*D* = 25 *L* = 25	2	17, Pb	*γ*	–	3.7	6.5% (^18^F)	415–680 keV	–	23%

*D* diameter, *L* length

^a^For cylindrincal detector

Although energy resolution is not a critical feature for blood counting in PET, the CZT detector used here achieved a relatively good value of 8% at 662 keV, better than any crystal-based solution. This energy resolution let efficient counting in dual-isotope studies.

A single detector module was used in the experiments reported here. This makes the device potentially very sensitive to background radiation. In this regard, the acquisition card and associated firmware were designed to accommodate a second detector module and to perform coincidence counting or photopeak summation [6]. This could further reduce the size and weight of the device as less shielding would be required to achieve the same MDA. Further modelling efforts will be conducted to devise the optimal combination of acquisition mode and shielding requirements to make the detector immune to background variations.

It is important to note that positrons have a non-null probability of reaching the CZT crystal, especially for emitters having an *E*_*max*_ larger than 1.2 MeV such as ^15^O (*E*_*max*_ = 1.735 MeV) in the geometry proposed here. Positrons with a lower energy will most likely be absorbed by the material located between the emission position and the CZT crystal (1 mm Al, 1.2 mm plastic and 0.8 mm air in our case). Positron events in CZT can lead to different outcomes, one of which is an energy deposition larger than 511 keV (if one or two annihilation photon interact afterwards in CZT). The other possibility, where both photons escape, produce an event which cannot be differentiated from a gamma interaction and contribute to the system deadtime. In all cases, the effect is systematic for a given geometry and isotope and is easily handled with calibration. Therefore, no problems are anticipated for the use of positron emitters other than ^18^F assuming proper calibration.

## Conclusions

Results obtained in this study demonstrate the feasibility of using a CZT-based detector to obtain the input function for PET or SPECT pharmacokinetic modeling. The large CZT detector used here (20 × 20 × 15 mm^3^) has the advantage of converting a relatively large fraction of incident gamma at 511 keV. Furthermore, the detector module has a compact design requiring less shielding than PMT-based solutions, provides a better energy resolution than crystal-based solutions and can be operated at room temperature. The MDA of the device was 2.5 kBq/ml for 3 s sampling duration, and it can be improved by a factor 3.2 by increasing the sampling time to 30 s for low activity measurements. The compact device was shown to be stable in time and robustness. The measurement of a TAC in a PET study confirms that the device is adequate for use in a clinical setting. Future work will include the development of an automatic blood packaging system so that additional biochemistry and calibration tests can be performed easily. This is especially important for radiopharmaceuticals requiring metabolite corrections.
